# Gastrointestinal symptoms before and during menses in healthy women

**DOI:** 10.1186/1472-6874-14-14

**Published:** 2014-01-22

**Authors:** Matthew T Bernstein, Lesley A Graff, Lisa Avery, Carrie Palatnick, Katie Parnerowski, Laura E Targownik

**Affiliations:** 1 Department of Internal Medicine, University of Manitoba, Winnipeg, Canada; 2 Department of Clinical Health Psychology, Faculty of Medicine, University of Manitoba, PZ350 - 771 Bannatyne Avenue, Winnipeg, MB R3E 3N4, Canada; 3 Department of Obstetrics and Gynecology, University of Manitoba, Winnipeg, Canada

## Abstract

**Background:**

Little is known as to the extent gastrointestinal (GI) complaints are reported by women around menses. We aimed to describe GI symptoms that occurred premenstrually and during menses in healthy women, and to specifically assess the relationship of emotional symptoms to GI symptoms around menses.

**Methods:**

We recruited healthy, premenopausal adult women with no indication of GI, gynecologic, or psychiatric disease who were attending an outpatient gynecology clinic for well-woman care. They completed a survey that queried menstrual histories and the presence of GI and emotional symptoms. We compared the prevalence of primary GI symptoms (abdominal pain, diarrhea, constipation, nausea, vomiting), as well as pelvic pain and bloating, in the 5 days preceding menses and during menses, and assessed whether emotional symptoms or other factors were associated with the occurrence of GI symptoms.

**Results:**

Of 156 respondents, 73% experienced at least one of the primary GI symptoms either pre- or during menses, with abdominal pain (58% pre; 55% during) and diarrhea (24% pre; 28% during) being the most common. Those experiencing any emotional symptoms versus those without were more likely to report multiple (2 or more) primary GI symptoms, both premenstrually (depressed p = 0.006; anxiety p = 0.014) and during menses (depressed p < 0.001; anxiety p = 0.008). Fatigue was also very common (53% pre; 49% during), and was significantly associated with multiple GI symptoms in both menstrual cycle phases (pre p < 0.001; during p = 0.01).

**Conclusions:**

Emotional symptoms occurring in conjunction with GI symptoms are common perimenstrually, and as such may reflect shared underlying processes that intersect brain, gut, and hormonal pathways.

## Background

Many women describe having gastrointestinal (GI) symptoms around their menses, yet little research has been done to quantify the prevalence or nature of these symptoms, or to consider associated factors. The handful of studies that have examined the occurrence of GI symptoms in relation to menses have enquired about a narrow range of symptoms (e.g. abdominal pain, bloating) or focused on individuals with established GI disorders [1-4]. A recent study by our group, in which women with inflammatory bowel disease were compared with a sample of healthy women on a range of upper and lower GI symptoms, found that perimenstrual GI symptoms were common both in women with and without inflammatory bowel disease [5].

Other research has examined emotional symptoms around menses, with many reporting that mood symptoms such as depression can be exacerbated premenstrually [6-8]. Strine et al. found that 19% of American women had menstrual complaints and those with menstrual complaints were more likely to also have mood symptoms [9]. However, it is unknown whether women who experience emotional symptoms around menses are any more likely to have concomitant GI symptoms. There is evidence that symptoms of depression and anxiety can influence the development and severity of GI symptoms within a variety of GI conditions such as inflammatory bowel disease and irritable bowel syndrome [10-12]. Nevertheless, there are no studies to date which have assessed whether there is a relationship between mood and GI symptoms around menses in women with no history of GI disease. Although physical symptoms are known to accompany premenstrual syndrome, there has been little research undertaken to document which specific physical symptoms occur, and to what extent they relate to depressive or other emotional symptoms [13].

Our prior report [5] focused on GI symptoms and menses in inflammatory bowel disease. In this report, we aimed to explore relationships among GI symptoms and emotional symptoms occurring in the context of menses in the cohort of healthy women. Discerning whether there is a relationship between emotional and GI symptoms at a particular point in the menstrual cycle (i.e., premenstrually and during menses) may help clarify why some are more prone to GI upset during this normal gynecological functioning. If there are interrelationships, this may provide direction for potential underlying pathways relevant to psychiatric, gastroenterological and gynecological functioning.

## Methods

### Participants

We recruited women from outpatient gynecology clinics in a large general hospital in Winnipeg, Canada. Individuals from a range of socioeconomic backgrounds attend these urban hospital clinics, given universal access for health care. Consecutive premenopausal women over the age of eighteen who were being seen for routine pelvic examinations and/or family counseling during a 3 month period in 2011 were invited to participate. Those with any known GI diagnoses, including inflammatory bowel disease, irritable bowel syndrome, celiac disease, those with active gynecological illness or symptoms such as endometriosis or dysfunctional uterine bleeding, and those with known active psychiatric disorders such as depression, panic disorder, schizophrenia and post traumatic stress disorder, were excluded. All participants completed a brief survey at the time of their clinic appointment.

All who took part were given information about the study, and provided their consent. The study was approved by the University of Manitoba Research Ethics Board.

### Questionnaire and design

A questionnaire was developed to assess the range of physical and emotional symptoms that might occur specifically in conjunction with the premenstrual and menses phases. As there were no validated measures that served that purpose, items were identified based on patient reports, literature review, and expert consensus from a team of gastroenterologists, gynecologists, and clinical psychologists. Items were included that briefly queried (a) menstrual history, including age at menarche, duration of menstrual cycle, history of painful periods, and use of medicinal contraception; (b) the presence of seven common GI symptoms, of which five - abdominal pain, constipation, diarrhea, nausea, and vomiting - were classified as primary GI symptoms. The remaining two - bloating and pelvic pain – were considered to be possibly gynecologic in origin in the context of the perimenstrual period, and thus classified as secondary GI symptoms; and (c) the presence of emotional symptoms (depressive symptoms, anxiety, ‘other’ emotional symptoms), and fatigue. The questionnaire was piloted with 20 women to assess clarity of the questions and completion time, and adjustments were made as needed based on feedback.

Participants were directed to consider their recent menstrual experiences (defined as the previous three menstrual cycles), and to report whether they had any of the GI or emotional symptoms, either in the five days before the onset of menses (pre-menstrual) and/or during menses. Since this was an exploratory study with the goal of a brief survey to readily engage participants, we did not ask more detailed questions at this point about the severity of any reported symptoms, nor did we include specific pain measures, for example. While aspects of medical history were reviewed to establish eligibility for participation, participants were not clinically evaluated regarding their medical history.

#### Outcomes

The main outcome of interest was the proportion of women reporting any of the specific individual gastrointestinal or emotional symptoms either prior to or during menses. We also calculated the proportion reporting 2 or more different primary gastrointestinal symptoms either prior to or during menses. Last, we stratified participants by the presence or absence of each of self-reported depressive symptoms, anxiety, fatigue, or history of painful menses, and determined the proportion of persons within each strata who reported any individual gastrointestinal symptoms as well as those who reported 2 or more gastrointestinal symptoms.

### Statistical analyses

Descriptive statistics were calculated detailing means, standard deviations, and proportions where appropriate. Differences in proportions, comparing prevalence of GI and emotional symptoms between the two menstrual phases, were assessed for statistical significance with Fisher’s exact test. Similarly, differences in proportions, comparing prevalence of GI symptoms for those with or without concurrent depressive symptoms, anxiety or fatigue, were each assessed for statistical significance using Fisher’s exact test. Finally, differences in proportions, comparing prevalence of GI symptoms for those with or without a history of painful menses, were assessed for statistical significance using Fisher’s exact test. P-values less than 0.05 were considered to be statistically significant.

## Results

**Figure 1:**
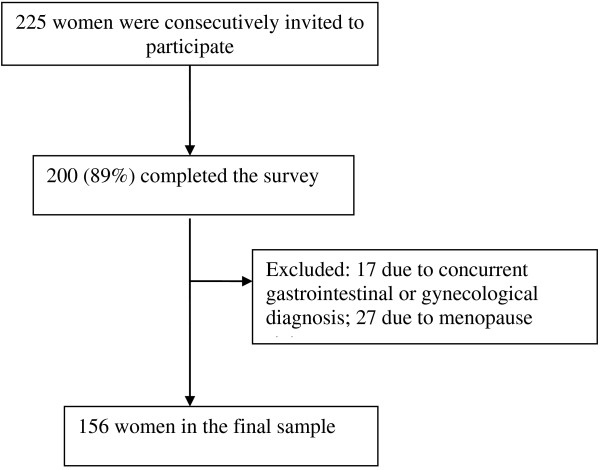
Flow diagram of participant recruitment in the outpatient clinics.

Of the 225 women invited to participate, 89% (n = 220) proceeded with the study. Of those, 156 healthy, premenopausal women met the eligibility requirements and provided completed data (Figure 1). The participants were between the ages of 18 and 55, with a mean age of 32.3 years (standard deviation 9.9 years). The mean age at menarche was 12.9 and the mean number of days of menses was 5.2. Just over 50% reported that their menses were typically painful (Table 1).

**Table 1:** Respondent characteristics related to menstrual history (n = 156)

**Menstrual information**	**Mean (SD)**
Age at first menses	12.9 (1.8)
Menses duration in days	5.7 (3.3)
	**n (%)**
Cycle duration	
<25 days	20 (13)
25–35 days	106 (68)
>35 days	30 (19)
History of painful menses	82 (53)
Using medicinal contraception	52 (33)

With regard to the prevalence of GI symptoms, nearly three-quarters of the sample (73%, n = 107) reported experiencing at least one of the primary GI symptoms premenstrually, and about two-thirds (69%, n = 107) reported at least one GI symptom during menses (Table 2). Thirty-one percent had multiple primary GI symptoms, either premenstrually or during menses. The prevalence of each GI symptom was similar across the two phases, with abdominal pain and diarrhea being the most common primary symptoms, and the secondary GI symptom of bloating being experienced most frequently overall. Depressive symptoms were the most common emotional symptoms reported in both the premenstrual and menses phases. A significantly higher proportion reported depressive symptoms premenstrually (32%) than during menses (21%, *P* = 0.028). Fatigue was fairly pronounced, with about half endorsing that symptom in either phase (53% premenstrual; 49% during menses).

**Table 2:** Proportion of women experiencing GI and emotional symptoms, comparing prevalence rates in premenstrual and menses phases

**Symptoms**	**Premenstrual**	**During menses**	**p-value**
**Primary GI symptoms**	%	%	
Abdominal pain	58	55	0.73
Diarrhea	24	28	0.44
Nausea	17	14	0.53
Constipation	15	10	0.08
Vomiting	2	3	0.72
Any primary symptoms	73	69	0.60
Multiple (≥2) primary symptoms	31	31	1.0
**Secondary GI symptoms**			
Bloating	62	51	0.07
Pelvic pain	49	46	0.73
Any primary or secondary GI symptoms	83	83	1.0
**Mood symptoms**			
Depressed mood	32	21	**0.028**
Anxiety	15	10	0.30
Other	23	15	0.08
Any emotional symptoms	47	31	**0.004**
**Fatigue**	53	49	0.50

Bolded p value denotes p < 0.05.

Tables 3, 4, 5 and 6 detail the frequency of GI symptoms in relation to emotional symptoms (Tables 3 and 4) and fatigue (Tables 5 and 6), separately considering the premenstrual and menstrual phases. Women experiencing depressive symptoms were significantly more likely to also report diarrhea, both before (36% vs 19%, p = 0.028) and during menses (50% vs 23%, p = 0.004). Those with anxiety symptoms were significantly more likely to report nausea both before (38% vs 13%, p = 0.006) and during menses (44% vs 11%, p = 0.002). Overall, individuals with emotional symptoms of either depression or anxiety, or those reporting fatigue were significantly more likely to experience multiple primary GI symptoms, both prior to the onset of menses and during menses (p < 0.02 for all comparisons).

**Table 3:** Proportion (%) of women with GI symptoms premenstrually, comparing those with or without emotional symptoms

	**Depressive symptoms**			**Anxiety symptoms**		
	**Yes**	**No**	**P value**	**Yes**	**No**	**P value**
	**n = 50**	**n = 106**		**n = 24**	**n = 132**	
**Primary GI symptoms**	%	%		%	%	
Abdominal pain	68	53	0.118	62	58	0.88
Diarrhea	36	19	**0.028**	42	21	**0.040**
Nausea	28	11	**0.012**	38	13	**0.006**
Constipation	24	11	0.056	25	14	0.27
Vomiting	4	1	0.40	8	1	0.062
Any primary symptom	86	67	**0.012**	75	73	1.0
Multiple (≥2) primary symptoms	46	24	**0.006**	54	27	**0.014**
**Secondary GI symptoms**						
Bloating	82	52	**<0.001**	71	60	0.43
Pelvic pain	76	36	**<0.001**	58	47	0.42

Bolded p value denotes p < 0.05.

**Table 4:** Proportion (%) of women with GI symptoms during menses, comparing those with or without emotional symptoms

	**Depressive symptoms**			**Anxiety symptoms**		
	**Yes**	**No**	**P value**	**Yes**	**No**	**P value**
	**n = 32**	**n = 124**		**n = 16**	**n = 140**	
**Primary GI symptoms**	%	%		%	%	
Abdominal pain	69	51	0.110	63	54	0.72
Diarrhea	50	23	**0.004**	50	26	0.074
Nausea	25	12	0.082	44	11	**0.002**
Constipation	19	7	0.085	13	9	0.94
Vomiting	6	2	0.187	13	1	0.053
Any primary symptom	81	66	0.144	81	68	0.42
Multiple (≥2) primary symptoms	59	23	**<0.001**	63	27	**0.008**
**Secondary GI symptoms**						
Bloating	78	44	**0.001**	69	49	0.186
Pelvic pain	72	39	**0.001**	69	43	0.064

Bolded p value denotes p < 0.05.

**Table 5:** Proportion (%) of women with GI symptoms premenstrually, comparing those with or without fatigue

	**Fatigue**		
	**Yes n = 83**	**No n = 73**	**P value**
**Primary GI symptoms**	%	%	
Abdominal pain	63	53	0.32
Diarrhea	34	14	**0.005**
Nausea	23	10	**0.032**
Constipation	19	11	0.185
Vomiting	4	0	0.51
Any primary symptom	78	67	0.148
Multiple (≥2) primary symptoms	43	16	**<0.001**
**Secondary GI symptoms**			
Bloating	77	44	**<0.001**
Pelvic pain	63	33	**<0.001**

Bolded p value denotes p < 0.05.

**Table 6:** Proportion (%) of women with GI symptoms during menses, comparing those with or without fatigue

	**Fatigue**		
	**Yes**	**No**	**P value**
	**(n = 48)**	**(n = 107)**	
**Primary GI symptoms**	%	%	
Abdominal pain	61	49	0.197
Diarrhea	34	23	0.113
Nausea	19	10	0.168
Constipation	13	6	0.179
Vomiting	5	0	0.054
Any primary symptom	72	66	0.478
Multiple (≥2) primary symptoms	41	21	**0.010**
**Secondary GI symptoms**	%	%	
Bloating	63	39	**0.003**
Pelvic pain	58	34	**0.004**

Bolded p value denotes p < 0.05.

Participants who had a history of painful menses in general were also significantly more likely to experience primary GI symptoms. In the premenstrual phase, those with a history of painful menses were more likely to report abdominal pain (73% v 42%, p = 0.0001), diarrhea (32% v 16%, p = 0.04), and nausea (27% v 5%, p = 0.0005) than those without painful menses. In the menstrual phase, those with a history of painful menses were more likely to report abdominal pain (73% v 34%, p < 0.0001) and nausea (24% v 3%, p = 0.0001), compared to those not reporting painful menses (data not shown).

## Discussion

In this study, we found the experience of one or more GI symptoms was very common for healthy women both before and during menses. Not surprisingly, abdominal pain was quite frequent, but around one-quarter of the women also experienced bowel habit disturbance in the form of diarrhea. GI symptoms occurred at a similar rate in both the premenstrual phase and during menses. However, there was a higher prevalence of depressed mood and fatigue premenstrually, compared to during menses. As well, GI symptoms occurred disproportionately more frequently with depressive or anxious emotional symptoms than when those were not present, both prior to and during menses. This significant co-occurrence was also observed for fatigue.

Studies that have assessed the prevalence of various GI symptoms perimenstrually have generally concluded that GI symptoms were more common for those with GI disorders than for healthy women [1,3,14,15]. It was evident in our study that GI symptoms were quite prevalent for healthy women as well, as over 70% experienced GI symptoms in conjunction with their menstrual cycle, even when potential gynecological symptoms such as bloating were excluded. Some studies focused just on menses, and when they included more than one phase, they tended to report more frequent GI symptoms during menses than other phases [3,15], although one of these studies reported on intensity but not the prevalence of GI symptoms [15]. Other prospective studies described abdominal pain, nausea, and bloating as the predominant GI symptoms, and found they tended to increase just before and during menstruation [1,16,17] consistent with our findings of a similar rate of GI symptom occurrence across the premenstrual and menses phases.

Bowel habit changes have not been as readily addressed, but Kane and colleagues [14] described equivalent rates of diarrhea (20%) and constipation (20%) premenstrually, and lower rates of altered bowel habit during menses (diarrhea 10%; constipation 2%) in their sample of healthy women. Two studies found that approximately one third of women experienced bowel habit changes during menses, with diarrhea being more common [1,4]. We also found diarrhea (24-28%) to be more common than constipation (10-15%), regardless of the menses phase. The lower rates for the Kane study might relate to their recruitment approach as they posted ads on a university campus, whereas the other studies, including ours, recruited from outpatient clinics offering routine gynecological care.

There has been little work to examine potential predictors of GI symptoms in relation to menses. Our exploratory study identified that depressed mood, anxiety and fatigue were each significantly more likely to be associated with primary GI symptoms. Similarly, women who had a history of painful menses were also more likely to experience GI symptoms perimenstrually. Previous work assessing the relationship between GI symptoms and both enduring personality traits and acute psychological symptoms with GI symptoms during menstruation did not find any significant relationship [1]. In that study, women who reported their GI symptoms were exacerbated during their menses did not differ in their psychological profiles from women who did not report these symptoms [1]. Keisner and colleagues reported a significant association between premenstrual depressive symptoms and a number of physical symptoms, of which GI symptoms were included [13].

Depression, pain, and gut motility may share similar pathophysiological mechanisms including serotonin as an important neurotransmitter mediating those symptoms [1]. A study that found women in the late luteal phase experienced reduced pain tolerance, using a cold pressor test, provides some evidence for somatic neural changes related to the timing within the cycle [18]. There has also been consideration of the effect of hormonal activity in local tissue, with a recent study suggesting that physical symptoms, including GI symptoms, may indicate sensitivity to reproductive steroids, and that concurrent psychological symptoms may reflect neurological sensitivity to these steroids, at a peak point in the menstrual cycle [19]. Prostaglandins may provide another pathophysiological link to understand the overlap between menstrual pain and gastrointestinal symptoms. Premenstrually, uterine prostaglandin production may mediate an inflammatory response characterized by pain, and during menses abnormally high levels of prostaglandins in menstrual fluid may induce abnormal uterine contractions and pain [17,20]. In the gut, prostaglandins can cause smooth muscle contractions, as well as reduced absorption and induced secretion of electrolytes in the small bowel, all of which may enhance gastrointestinal pain and diarrhea [21]. It is not known whether uterine prostaglandins are transported to the gut, or whether parallel changes in uterine and GI smooth muscle prostaglandin levels occur during menses [17]. Further study will be necessary to determine pathophysiological mechanisms for mood changes within the cycle as well and the direction of the relationship of these changes between brain and GI function.

While these findings are preliminary, they suggest that clinicians should be aware of the heightened potential for co-occurring gastrointestinal and emotional symptoms perimenstrually, and could consider providing information to their patients to help normalize the experience. If the GI symptoms become troubling or problematic, it may be useful to consider prophylactic steps to alleviate the symptoms through use of medication or behavioral approaches, parallel to the approach used to manage gynecological symptoms during menses (e.g., analgesic medication for dysmenorrhea).

There are limitations to the study. It was exploratory in nature, aiming to assess the presence of GI and emotional symptoms perimenstrually using participant observation, with minimal participant burden. There were no validated scales that included all the symptoms of interest, so a brief history and symptom measure was developed for the study. This had the benefit of assessing the variables of interest using the same response scale, for ready comparison. However, the validity of the symptom measure was not established, and further, the duration and severity of symptoms could not be determined as the measure simply assessed presence/absence of symptoms. Subsequent investigation of potentially relevant variables identified in this preliminary study, such as depression, anxiety, and pain, should include validated measures. Second, participants reported their perimenstrual symptoms retrospectively, which increases the likelihood of recall bias. Nevertheless, it was a relatively brief recall period of the recent 3 ‘samples’ of their cycle, and it has been shown that asking about very recent events helps to minimize recall bias [22]. In addition, the cross-sectional design of the study did not allow for any conclusions regarding direction of influence. A prospective approach using daily symptom diaries would be optimal for future studies. Finally, though we specifically recruited healthy premenopausal women, the experiences of healthy women presenting for care in a gynecology clinic may not be broadly generalizable to premenstrual women, many of whom do not seek or have access to regular gynecologic care.

## Conclusions

In conclusion, the occurrence of GI symptoms in conjunction with the premenstrual and menses phases is fairly common, and is disproportionately more likely if there are also accompanying emotional symptoms. These co-occurring experiences may reflect underlying common mechanisms which can provide direction for symptom relief. Given the exploratory nature of the study, prospective work tracking GI and emotional symptom severity in the context of the menses phases will be important as a next step. It will be useful to discern whether GI symptoms contribute to the mood disorder that affects many women prior to and during menses or alternatively, whether depressive and anxiety symptoms are predominately impacting on GI symptoms, as these relationships may have implications for managing the symptoms therapeutically.

## Competing interests

Laura E Targownik is supported by a Canadian Institutes of Health Research New Investigator Award. The authors declare they have no competing interests.

## Authors’ contributions

MT was involved in the concept, design, data acquisition, analyses and interpretation of data, manuscript draft preparation, and revisions for critical content. LG was involved in the concept, design, analyses and interpretation of data, manuscript draft and revisions for critical content. LA was involved in the concept, design, data acquisition, manuscript draft preparation and revisions for critical content. CP was involved in the data acquisition, analyses and interpretation of data, manuscript draft preparation and revisions for critical content. KP was involved in the data acquisition, manuscript draft preparation and revisions for critical content. LT was involved in the analyses and interpretation of data, manuscript draft and revisions for critical content. All authors read and approved the final manuscript.

## Pre-publication history

The pre-publication history for this paper can be accessed here:

http://www.biomedcentral.com/1472-6874/14/14/prepub
